# Hippocampal Proteomics Reveals the Novel Molecular Profiling of Postnatal Lead (Pb) Exposure on Autism-like Behaviors

**DOI:** 10.3390/toxics13060465

**Published:** 2025-05-31

**Authors:** Li Liu, Xulan Zhou, Zihan Ma, Ruming Liu, Yuhan Zhang, Yaqi Wang, Yiwen Liu, Xiaochun Xia, Juan Wang

**Affiliations:** 1 School of Public Health, Fujian Medical University, Fuzhou 350122, China; 990712@fjmu.edu.cn; 2 Department of Public Health and Medical Technology, Xiamen Medical College, Xiamen 361023, China; zxl@xmmc.edu.cn (X.Z.); zihan_ma0317@163.com (Z.M.); ruming_liu0304@163.com (R.L.); liu10300225@163.com (Y.L.)

**Keywords:** autism spectrum disorder (ASD), lead (Pb), postnatal, hippocampus, proteomics

## Abstract

Autism spectrum disorder (ASD) is a multifactorial neurodevelopmental disorder, with lead (Pb) exposure increasingly linked to its risk. However, the molecular mechanisms linking Pb to ASD remain poorly understood. This study established a postnatal Pb-exposed mouse model and employed the three-chamber social test and the marble-burying test to assess ASD-like behavioral phenotypes. The Pb levels in both blood and the hippocampus were quantified, and hippocampal neurons were assessed for morphological alterations. Moreover, a Tandem Mass Tag (TMT)-based quantitative proteomics approach was applied to elucidate the underlying mechanisms. Neurobehavioral experiments revealed Pb-exposed C57BL/6 offspring exhibited reduced social interaction and novelty preference along with increased repetitive marble-burying behavior. The Pb levels in both the blood and hippocampus of Pb-treated mice were significantly elevated compared with those of control animals. Postnatal Pb exposure resulted in a reduction in the neuronal numbers and disorganized neuronal arrangement in the hippocampus. A total of 66 proteins were identified as being differentially expressed after postnatal Pb exposure. Among them, 34 differentially expressed proteins were common in both Pb exposure groups, with 33 downregulated and 1 upregulated. Bioinformatic analysis revealed multi-pathway regulation involved in Pb-induced neurodevelopmental disorders, including dysregulation of synaptic signaling, abnormal activation of neuron apoptosis, and neuroinflammation. Notably, the SYT10/IGF-1 signaling pathway may play a potential key role. These findings enhance understanding of Pb-induced autism-like behaviors, providing novel proteomic insights into the etiology of ASD.

## 1. Introduction

Autism spectrum disorder (ASD) encompasses a group of neurodevelopmental conditions characterized by impaired social interaction coupled with repetitive behaviors. The Centers for Disease Control and Prevention (CDC) reported that 1 in 36 children in the United States was diagnosed with ASD in 2020 [1]. The prevalence of ASD in Chinese children is estimated at 7‰ (almost 1/142) [2]. Boys are, on average, four times more likely to be diagnosed with ASD than girls. ASD accounts for 11.5 million all-age disability-adjusted life-years (DALYs), with an age-standardized DALY rate of 147.6 per 100,000 people. Notably, ASD was ranked among the top 10 causes of non-fatal health burden for people under 20 years old [3]. Due to its high prevalence and substantial socioeconomic costs, more attention should be paid to the etiology of ASD for the purposes of early screening and intervention.

While both genetic and environmental factors likely contribute to the development of ASD, research has suggested that lead (Pb) is recognized as an important environmental risk factor for ASD [4,5,6,7]. A large number of studies have demonstrated that Pb levels are higher in children with than in those without ASD [8,9,10,11,12,13]. Our previous study showed that there is a positive correlation between Pb levels and symptom severity in children with ASD (*p* < 0.001) [14], which is consistent with the findings of earlier research [15,16]. Childhood neurodevelopment is marked by critical periods that determine brain plasticity. It has been shown that exposure to heavy metals during 10 to 30 weeks after birth increases the risk of ASD occurrence [17]. Compared with non-ASD control twin pairs, Pb levels in ASD discordant twins were 1.5 times higher at 15 weeks postnatally [18]. Additionally, Zhou et al. [19] reported that the postnatal period represents a sensitive window for perturbations in the methylation of the promoter of methyl-CpG-binding protein 2 (MECP2), a gene strongly implicated in ASD. Hence, identifying the potential threats of Pb, particularly postnatal exposure, to childhood neurodevelopment is crucial for exploring the etiology of ASD and safeguarding children’s health. Exposure to Pb has recently been associated with an increase in the levels of inflammatory cytokines, proinflammatory markers, and immunoglobulins, which may contribute to the development of autism-like symptoms in a mouse model of autism [20,21,22]. Pb can induce ASD-like behavior by triggering oxidative stress [23]. These findings highlight the need for in-depth studies on the mechanisms underlying the effects of Pb exposure on ASD development. However, the relevant research information remains limited. This study aimed to systematically explore the association between postnatal Pb exposure and the development of ASD as well as its underlying molecular mechanisms.

Quantitative proteomic analysis helps us to develop a more thorough understanding at the protein level for neurotoxicity, and some of the key proteins identified can also potentially be used as biomarkers in epidemiologic studies [24,25,26]. Using TMT-based quantitative proteomics, the synaptic proteomes across five mouse models for ASD were analyzed, and significant dysregulations were identified in multiple cellular and molecular pathways at the synaptic level, including oxidative phosphorylation and Rho family small GTPase signaling [27]. In this study, we employed TMT-based proteomics to screen for protein profiling in male offspring subjected to postnatal Pb exposure and investigate their potential connections to ASD. The aim was to further elucidate the underlying molecular mechanisms linking Pb exposure and ASD and to screen the potential intervention targets.

## 2. Materials and Methods

### 2.1. Establishment of Animal Models and Grouping

C57BL/6 mice were purchased from the Experimental Animal Centre of Xiamen University (Xiamen, China). The mice were housed with laboratory chow and distilled water and individually housed in an ambient temperature (20 ± 2 °C) and relative humidity (50 ± 10%) controlled environment on a 12 h:12 h light/dark cycle. Mice had free access to food and water. All animal experiments complied with the Animal Research: Reporting of In Vivo Experiments (ARRIVE) guidelines. The experiments were conducted in accordance with the National Institutes of Health Guide for the Care and Use of Laboratory Animals and were approved by the Medical Ethics Committee of Xiamen Medical College (Approval No. 2021041311).

**Figure 1:**
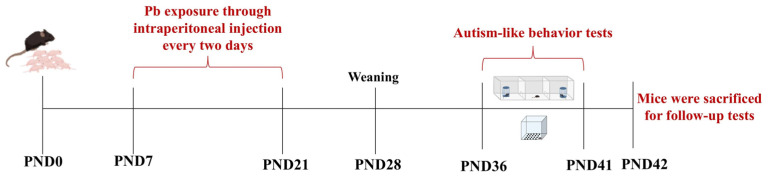
Visual representation of the comprehensive research protocol timeline.

Male and female mice (20 ± 2 g, 1: 2) were mated overnight, and the vaginal secretion was collected the next morning. Pregnant females were raised independently. The day of birth was considered postnatal day 0 (PND0) (Figure 1). Male offspring were randomly divided into a control group, a low-Pb group (15 mg/kg bodyweight PbAc), and a high-Pb group (30 mg/kg bodyweight PbAc). Each group included 15 male offspring. The experimental design and the Pb exposure doses were based on a previous study [28,29]. Male offspring were intraperitoneally injected with PbAc dissolved in saline solution every two days from PND7 until PND21 to evaluate the effect of Pb during critically developmental windows. The control group received saline solution (sterile 0.9% NaCl). After Pb exposure, the male offspring were housed as normal until used for follow-up experiments.

### 2.2. Three-Chamber Social Test

**Figure 2:**
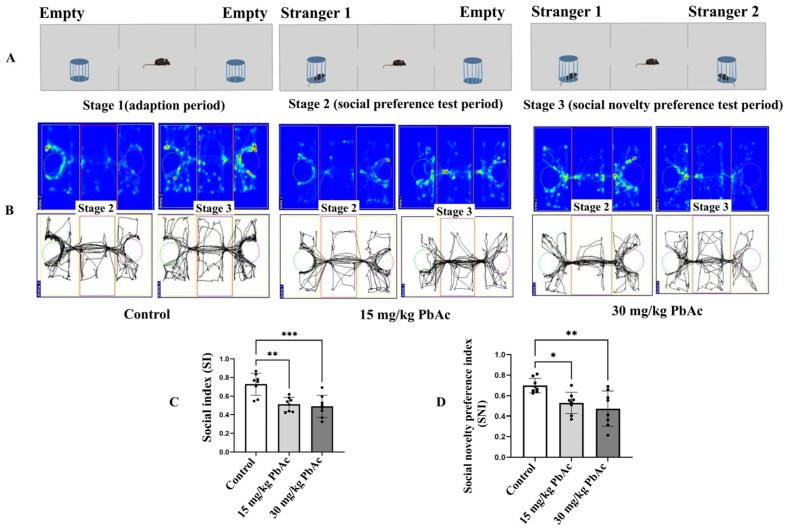
Influence of postnatal Pb exposure on social deficits in offspring mice (*n* = 8/each group). (**A**) Experimental protocol of three-chamber social test. (**B**) Track of male offspring in the three-chamber test. The traces on the left side of each group represent the behavior activities in stage 2, while those on the right side represent the behavior activities in stage 3. In stage 2, the green circle means a mouse, and the purple circle means an empty cage; while in stage 3, the green circle means a familiar mouse, and the purple circle means a strange mouse. (**C**) Quantification of the social index and (**D**) the social novelty preference index in the three-chamber test showed decreased social behaviors of Pb-exposed offspring. * *p* ≤ 0.05, ** *p* ≤ 0.01, *** *p* ≤ 0.001.

The three-chamber social test was conducted to evaluate the sociability of male offspring. The test was performed as previously described [30,31] with minor modifications. The apparatus consisted of a box with three chambers (length 65 cm, width 45 cm, height 20 cm) with small rectangular openings allowing access to each chamber. A wire cage was placed in the middle chamber. As shown in Figure 2A, in stage 1 (adaption period), an empty wire cage was placed in both the left and right chambers. In stage 2 (social preference test period), a never-before-encountered mouse (Stranger 1) was placed under the wire cage in the left chamber, while the cage in the right chamber remained empty. Finally, in stage 3 (social novelty preference test period), a second never-before-encountered mouse (Stranger 2) was placed under the wire cage in the right chamber. In each stage, the test mouse was placed in the middle chamber and allowed to freely explore for 10 min. The time spent exploring the empty wire cage or the one with another mouse was measured, and the social index (SI) and social novelty preference index (SNI) were subsequently calculated. Data acquisition and analysis were performed using SMART software (Version 3.0.06).

### 2.3. Marble-Burying Test

As detailed in previous studies [32,33], the marble-burying test was used for the assessment of restricted repetitive behaviors in Pb exposed male offspring mice. The mouse was placed in a plastic container (length 40 cm, width 40 cm, height 40 cm) filled with clean woodchip bedding to a height of 5 cm. Twenty glass marbles (previously washed with 90% alcohol) were placed equidistant in the testing area. After the acclimation period, mice were allowed to freely bury marbles for 30 min. At the end of the test, the number of buried marbles was counted. Marbles covered at least two-thirds by bedding were considered buried. After each experiment, the marbles were thoroughly cleaned, and new bedding was placed in the testing chamber.

### 2.4. Pb Level Determination in Blood and Hippocampus

Pb level determination in blood and the hippocampus was performed as previously described [34,35] with minor modifications. At the end of the social tests, fresh blood and hippocampal tissue were immediately collected. Blood (0.25 mL) or hippocampal tissue (0.1 g) was placed in 5 mL of 65% nitric acid at room temperature for 6 h, followed by digestion with a microwave digestion system (MARSXpress, CEM Corporation, Matthewss, NC, USA). Finally, the Pb level was detected using inductively coupled plasma mass spectrometry (ICP-MS; Agilent 7500cx, Palo Alto, CA, USA).

### 2.5. Histopathological Examination

To observe neuronal changes in the hippocampus of mice in the three groups, the hippocampus was collected on the day after the behavioral tests concluded. The hippocampus was washed with normal saline and immediately fixed in 4% paraformaldehyde. The tissue was dehydrated, embedded in paraffin (Leica AG, Frankfurt, Germany), sliced into 5 mm-thick sections, and stained with hematoxylin and eosin (H&E). The hippocampal subregions, namely, the cornu ammonia 1 (CA1), CA2, CA3, and dentate gyrus (DG), play distinct roles in socialization, learning, and memory. The sections were examined in detail under a light microscope (Olympus Co., Tokyo, Japan) to identify morphological changes in any of the above-mentioned regions.

The hippocampal neurons were quantitatively analyzed using Image J software (Version 1.53e). Hippocampal neurons were identified by recognizing blue-purple stained cell nuclei and manually counted in each visual field. Two visual fields were observed for each section, with a total of six visual fields observed in each group. Finally, the total numbers of hippocampal neurons in each group were statistically analyzed.

### 2.6. TMT-Based Quantitative Proteomics

#### 2.6.1. Protein Sample Preparation

Hippocampal protein was prepared as previously reported [36,37,38]. For protein extraction, the samples were placed in lysis buffer and homogenized using an automatic grinder. The supernatant was collected by centrifugation at 25,000× *g* for 15 min at 4 °C. Then, protein samples (100 μg) were reduced, alkylated, and precipitated by adding 5× the volume of chilled acetone and placing at −20 °C for 2 h. The precipitated proteins were resolved with SDS-free lysis buffer and centrifugated at 25,000× *g* for 15 min at 4 °C. The collected protein was quantitated using the Bradford assay, and protein integrality was assessed by SDS-PAGE. Precipitated proteins were digested for 4 h at 37 °C in 100 μL of TEAB buffer containing sequence-grade trypsin at a trypsin-to-protein mass ratio of 1:20. Enzymatic cleavage was terminated by adding 5 μL of 10% formic acid, and the collected peptide fractions were freeze-dried.

#### 2.6.2. TMT Labeling

TMT labeling was conducted to reconstitute and process peptides based on the protocol of TMT Multiplex Kit (Thermo Fisher, Waltham, MA, USA). The TMT reagent was warmed to room temperature; then, 41 μL (0.8 mg/tube) acetonitrile was added to the TMT reagent and shaken thoroughly. Digested peptide (100 μg) was mixed with 41 μL of TMT reagent. The reaction was terminated by shaking followed by centrifugation and incubation at room temperature for 2 h. TMT-labeled mixed peptides were fractionated using liquid chromatography (LC-20AB, Shimadzu, Kyoto, Japan) coupled with a Gemini C18 column (250 mm × 4.6 mm × 5 μm). In the binary solvent system, the mobile phase A contained 5% acetonitrile (pH 9.8), whereas mobile phase B contained 95% acetonitrile (pH 9.8). The dried peptide samples were reconstituted with mobile phase A and injected. LC separation was performed at a flow rate of 500 µL/min by the following gradients: 5% B for 10 min, 5–35% B for 40 min, 35–95% B for 1 min, 100% B for 3 min, and 5% B for 10 min. The elution peak was monitored at a wavelength of 214 nm, one component was collected per minute, and the samples were combined according to the chromatographic elution peak map to obtain 20 fractions, which were then vacuum-dried.

#### 2.6.3. LC-MS/MS Analysis

Dried polypeptides were reconstituted in mobile phase A (2% acetonitrile, 0.1% formic acid) and centrifuged at 20,000× *g* for 10 min. One microliter of supernatant was injected into a Thermo UltiMate 3000 UPLC. Each sample was separated at a flow rate of 300 nL/min using the following effective gradient: 5% mobile phase B (98% acetonitrile, 0.1% formic acid) in 5 min, 5–25% B over 40 min, 25–35% B over 5 min, 35–80% B over 2 min, 80% B over 2 min, and 5% B for 6 min. The peptides separated by liquid chromatography were ionized using a nanoESI source and then analyzed in a Q-Exactive HF X tandem mass spectrometer (Thermo Fisher Scientific, San Jose, CA, USA) in data-dependent acquisition (DDA) mode. The main parameters were set as follows: ion source voltage, 2 kV; MS1 scanning range, 350–1500 *m/z*; resolution, 60,000; MS2 starting *m/z*, 100, with a resolution of 15,000. MS2 fragmentation was performed on ions with charges ranging from 2+ to 6+, selecting the top 20 parent ions with peak intensities exceeding 20,000. High-energy collisional dissociation (HCD) was used for ion fragmentation with Orbitrap detection. The dynamic exclusion time was set to 30 s, and the automatic gain control was set to 1E5 for MS1 and 2E4 for MS2.

#### 2.6.4. Processing and Analysis of the Spectral Data

The raw files obtained from LC-MS/MS were searched against the UniProt Protein Database (version 2.4, Thermo Scientific, San Jose, CA, USA) using the following parameters: Precursor ion mass tolerance: 20 ppm; Fragment ion mass tolerance: 20 ppm; Maximum missed cleavages: 2. The false discovery rate (FDR) was set at 1%. DEPs were identified based on a fold change (FC) greater than 1.5 between Pb-treated group and control group with a *p*-value less than 0.05. The DEPs were annotated through Gene Ontology (GO) and Kyoto Encyclopedia of Genes and Genomes (KEGG) databases. In GO annotation, the DEPs were classified into subcategories, namely, biological process (BP), cellular component (CC), and molecular function (MF). The functions of DEPs were explored through GO and KEGG enrichment analysis. A heatmap was plotted on an online platform (https://www.bioinformatics.com.cn, last accessed on 10 December 2024) for data analysis and visualization. Protein–protein interaction (PPI) was completed by Markov Clustering (MCL) with the inflation parameter of more than 3 by using the STRING database (https://cn.string-db.org/, last accessed on 5 April 2025).

### 2.7. Western Blotting Analysis

To further investigate the potential targets for ASD, we analyzed the protein expression levels of key synapse-related proteins identified in proteomics, including synaptotagmin-10 (SYT10) and syntaxin-16 (STX16). Additionally, the expression level of insulin-like growth factor 1 (IGF-1), a downstream signaling factor of SYT10, was assessed via Western blotting.

Total protein was extracted from hippocampal tissue. Equal amounts of protein were separated by sodium dodecyl sulfate–polyacrylamide gel electrophoresis (SDS-PAGE), transferred to a polyvinylidene fluoride (PVDF) membrane via wet transfer, blocked, incubated with primary antibodies against SYT10 (1:2000, BioSource, Muskego, WI, USA), STX16 (1:1000, Invitrogen, Waltham, MA, USA), IGF-1 (1:1000, Cell Signaling Technology, Danvers, MA, USA), glyceraldehyde-3-phosphate dehydrogenase (GAPDH, 1:10,000, Proteintech, Rosemont, IL, USA), or β-actin (1:10,000, Abbkine, Wuhan, China) at 4 °C overnight, and then with peroxidase-conjugated goat anti-rabbit or anti-mouse immunoglobulin G secondary antibody (1:10,000, Abcam, Cambridge, UK) at room temperature for 1 h. After exposure using an ECL reagent, the bands were visualized and imaged using the ECL imaging system (Bio-Rad, Berkeley, CA, USA).

### 2.8. Statistical Analysis

All results for each group were expressed as means ± SEM. Differences among groups were analyzed using one-way or two-way ANOVA, followed by Tukey’s multiple-comparison test. Post hoc tests were performed when F in ANOVA achieved the necessary level of statistical significance (*p* < 0.05) and there was no significant variance in homogeneity. Student’s *t*-tests were used to compare two sets of data.

## 3. Results

### 3.1. Postnatal Pb Exposure Induced Autism-like Behaviors in Mice

The three-chamber social test was employed to elucidate the social behavior deficits in mice exposed postnatally to Pb (Figure 2A). In the sociability stage of the test, control mice spent more time in the chamber containing the stranger than in the empty chamber. In contrast, mice in both the 15 and 30 mg/kg PbAc groups showed no significant difference for either chamber (Figure 2B). Accordingly, mice in the Pb exposure groups demonstrated a significant decrease in the SI and the SNI compared with those in the control group (*p* < 0.01, Figure 2C,D).

**Figure 3:**
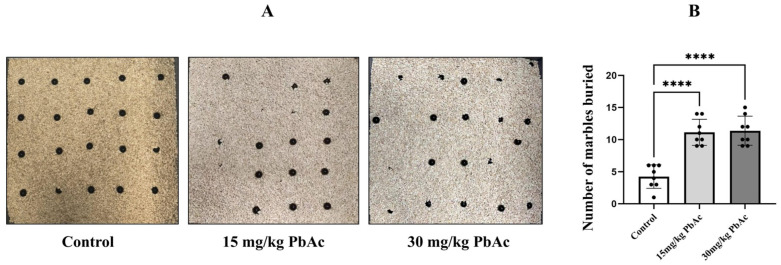
Influence of postnatal Pb exposure on repetitive behaviors in offspring mice (*n* = 8/each group). (**A**) Experimental demonstration diagram of the marble-burying test. (**B**) The Pb-exposed mice induced a significant increase in the number of buried marbles compared with the control mice. **** *p* ≤ 0.0001.

The marble-burying test was employed to quantify restricted repetitive behaviors (Figure 3A). Compared with control animals, Pb-treated mice showed a significant increase in the number of marbles buried (Figure 3B, *p* < 0.0001). These findings imply that mice exposed to Pb during the postnatal period display social deficits and repetitive behaviors, which are characteristic features of an ASD-like phenotype.

### 3.2. Pb Levels in Blood and the Hippocampus

**Figure 4:**
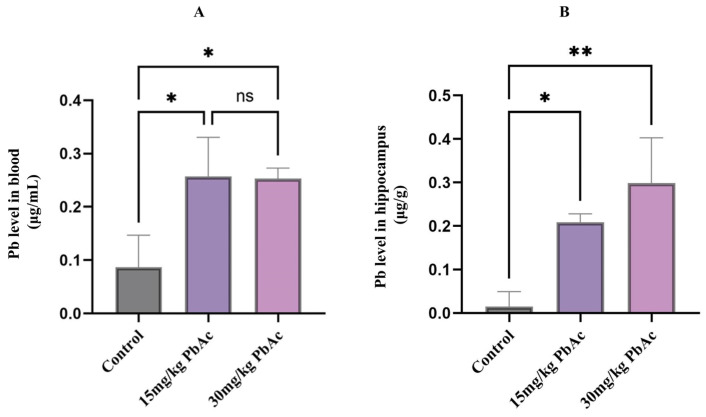
Pb levels in both the blood and hippocampus of offspring mice followed by Pb exposure (*n* = 3/each group). (**A**) Blood Pb levels. (**B**) Hippocampus Pb levels. * *p* ≤ 0.05, ** *p* ≤ 0.01. “ns” means no significant difference.

To assess the validity of the model established in this study, the Pb levels in blood and the hippocampus of mice were analyzed. The results indicated that blood Pb levels in the Pb-exposed groups were significantly elevated compared with those in the control group (*p* < 0.05, Figure 4). However, there was no significant difference in blood Pb levels between the Pb treatment groups (15 and 30 mg/kg PbAc; *p* > 0.05, Figure 4A). Additionally, the Pb levels in the hippocampal tissue of Pb-treated groups (15 and 30 mg/kg PbAc) were significantly higher than those in the control group (*p* < 0.01, Figure 4B).

### 3.3. Postnatal Pb Exposure Induced Morphological Alterations in the Hippocampus

**Figure 5:**
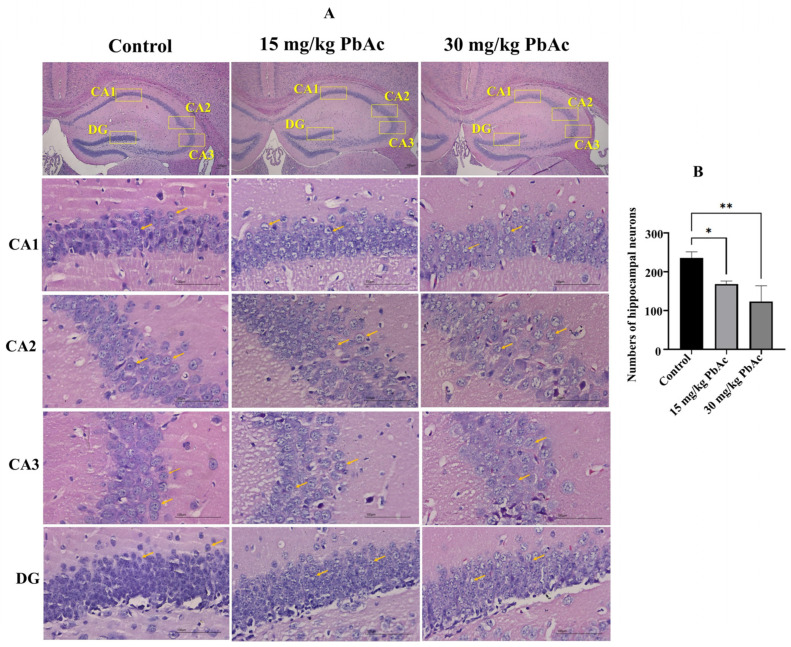
Postnatal Pb exposure induced morphological changes in the hippocampus of offspring mice (*n* = 3/each group). (**A**) H&E staining analysis. Low-magnification images (×100); high-magnification images (×400). Orange arrows represent neurons. (**B**) The neuron numbers in the hippocampus. * *p* ≤ 0.05, ** *p* ≤ 0.01.

We employed H&E staining to detect morphological changes in hippocampal subregions. In the control group, neurons in the CA1, CA2, CA3, and DG regions of the hippocampus were arranged in an orderly manner, with well-defined layers, distinct nucleoli, pale red cytoplasm, and no apparent pathological alterations (Figure 5A). In contrast, in the Pbexposed groups, there was a significant reduction in the number of hippocampal neurons (Figure 5B), the neurons showed a disordered arrangement, and the boundary with the surrounding tissues was unclear. Moreover, Pb exposure led to neuronal shrinkage and hyperchromatism, nuclear condensation, and a decrease in the number of nuclei. Nuclear condensation was most pronounced in the 30 mg/kg PbAc group, and there were fewer nuclei in the 30 mg/kg PbAc group than in the 15 mg/kg PbAc group, indicative of more extensive pathological changes.

### 3.4. Postnatal Pb Exposure Affected the Proteome Profiling

**Figure 6:**
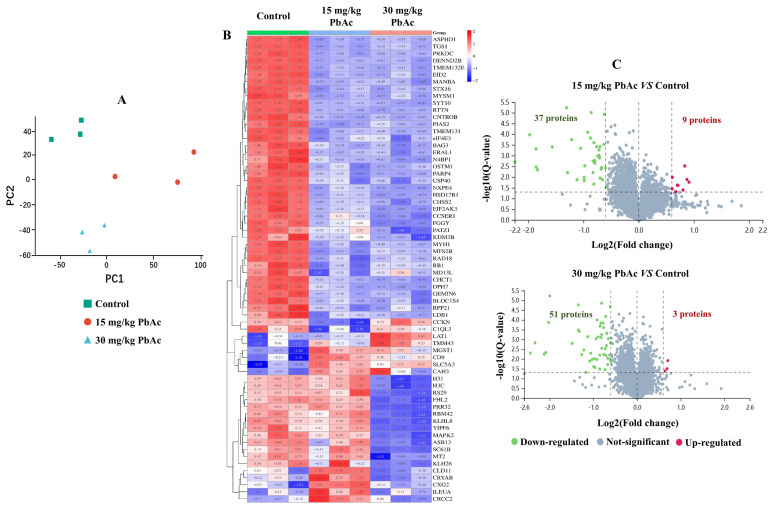
Differentially expressed proteins (DEPs) identified by TMT-based proteomic technology. (**A**) Principal component analysis (PCA). (**B**) Heatmap of DEPs. (**C**) Volcano plot of DEPs. The dots in red represent significantly upregulated DEPs, dots in green represent significantly downregulated DEPs, and dots in gray represent proteins with no significant difference in expression levels.

**Figure 7:**
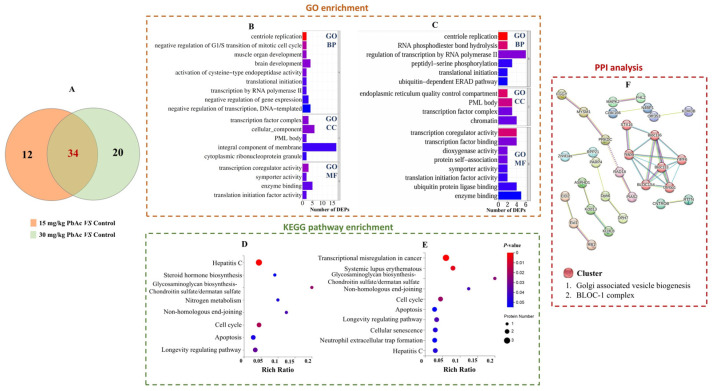
Bioinformatic analysis of DEPs. (**A**) Venn diagram of DEPs in Pb-exposed groups vs. control. (**B)** The top-ranked GO terms for a minimum of two DEPs in 15 mg/kg PbAc group. (**C**) The top-ranked GO terms for a minimum of two DEPs in 30 mg/kg PbAc group. (**D**) The significant enriched KEGG pathways in 15 mg/kg PbAc group. (**E**) The significant enriched KEGG pathways in 30 mg/kg PbAc group. (**F**) Protein–protein interaction (PPI) network diagram of target proteins.

To further elucidate the underlying molecular mechanisms linking postnatal Pb exposure and ASD-like behaviors, TMT-based proteomic analysis was employed to detect protein expression (*n* = 3/each group). A total of 7900 proteins were identified and quantified by referencing the *Mus musculus* database. Principal component analysis (PCA) of the proteomic profiling results indicated that there were distinct overall differences among the control, 15 mg/kg PbAc, and 30 mg/kg PbAc groups, with samples clustering within each respective group (Figure 6A). Compared with the control group, a total of 66 proteins were identified as being differentially expressed after postnatal Pb exposure, and then, a cluster analysis was performed as described by Tang et al. [39] (Figure 6B, Table S1). A total of 46 DEPs were identified in the 15 mg/kg PbAc group (37 downregulated and 9 upregulated), while 54 DEPs (51 downregulated and 3 upregulated) were detected in the 30 mg/kg PbAc group (Figure 6C). Among the DEPs identified in both Pb exposure groups, 34 were common to both groups (Figure 7A). Of these, 33 were downregulated and 1 was upregulated (Table S1).

### 3.5. Postnatal Pb Exposure Triggered Multi-Pathway Alterations

GO functional enrichment analysis was performed to identified DEPs. Among the 46 DEPs in the 15 mg/kg PbAc group, the top-ranked GO terms in the biological process (BP) category were mainly concentrated on the negative regulation of transcription and brain development. Furthermore, a total of 17 DEPs were annotated as integral components of the membrane in the cellular component (CC) category, while in the molecular function (MF) category, the top-ranked GO terms encompassed enzyme binding and transcription coregulator activity (Figure 7B). In the 30 mg/kg PbAc group, meanwhile, the top-ranked GO terms in the BP category were concentrated on the regulation of transcription by RNA polymerase II and peptidyl-serine phosphorylation. Within the CC category, the preeminent GO terms were centered around chromatin, transcription factor complex, and PML body. For MF, the top-ranked GO terms encompassed activities related to enzyme binding, ubiquitin protein ligase binding, transcription factor binding, and transcription coregulator activity (Figure 7C).

KEGG pathway enrichment analysis was used to identify major biochemical metabolic pathways and signal transduction pathways associated with the above DEPs. Significant enrichment of signaling pathways was observed in both Pb-exposure groups, with 8 and 10 pathways identified in the 15 mg/kg PbAc group and in the 30 mg/kg PbAc group (Figure 7D,E), respectively. Among them, six enriched KEGG signaling pathways—glycosaminoglycan biosynthesis-chondroitin sulfate/dermatan sulfate, non-homologous end-joining, cell cycle, apoptosis, longevity regulating pathway, and hepatitis C—were common to both Pb exposure groups. In addition, two signaling pathways were specifically enriched in the 30 mg/kg PbAc group, namely, transcriptional dysregulation in cancer and neutrophil extracellular trap formation. These results suggested that there are multiple pathways involved in Pb-induced ASD-like behaviors.

To further explore the connections among these pathways, a total of 66 DEPs were submitted to the STRING tool and constructed into an interaction network. As shown in Figure 7F, there was a close cluster between biogenesis of lysosome-related organelles complex 1 subunit 4 (BLOC1S4), syntaxin-16 (STX16), and Yip1 domain family member 6 (YIPF6), which are involved in Golgi-associated vesicle biogenesis and/or the BLOC-1 complex.

### 3.6. Postnatal Pb Exposure Decreased the Protein Levels of SYT10, STX16, and IGF-1

**Figure 8:**
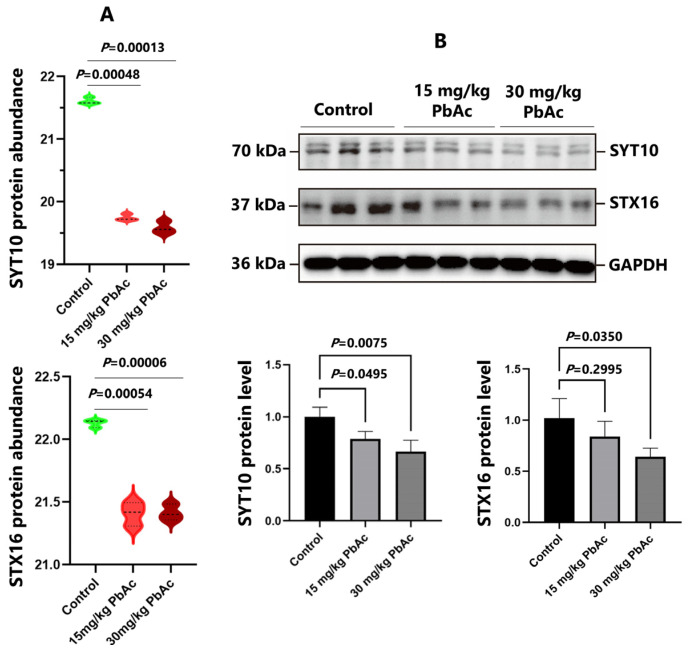
Postnatal Pb exposure inhibited the expression of SYT10 and STX16. (**A**) The expressed abundance of SYT10 and STX16 was detected with TMT-based proteomic analysis. (**B**) The protein level of SYT10 and STX16 was detected with Western blotting analysis.

**Figure 9:**
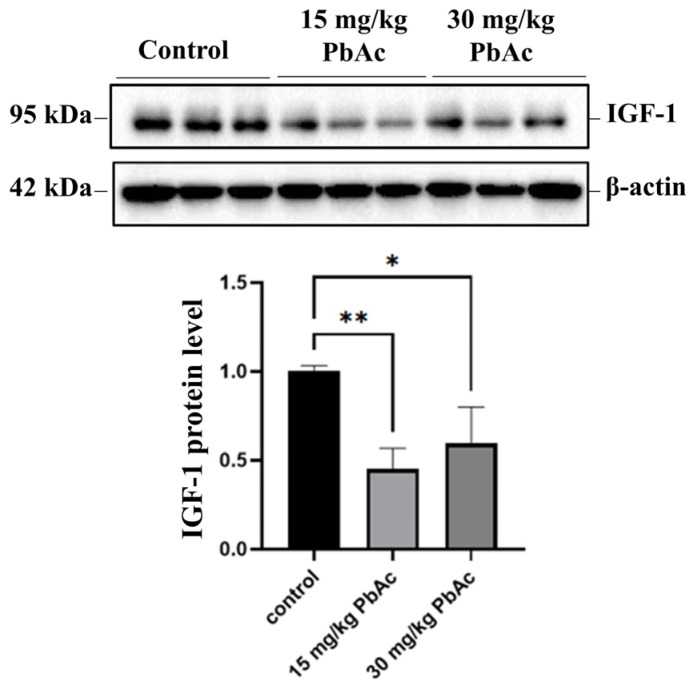
Postnatal Pb exposure inhibited the expression of IGF-1. * *p* ≤ 0.05, ** *p* ≤ 0.01.

Western blotting analysis was performed to quantify the protein levels of SYT10, STX16, and IGF-1 in the hippocampus after postnatal Pb exposure (*n* = 3/each group). Pb exposure resulted in a significant downregulation in protein levels of SYT10 and STX16, which were consistent with those obtained in the proteomic analysis (Figure 8). Meanwhile, the protein expression of IGF-1 was significant downregulated in both the 15 mg/kg PbAc group and the 30 mg/kg PbAc group (Figure 9).

## 4. Discussion

The pathogenesis of ASD remains elusive, which impedes the development of therapeutic and preventive strategies. Several lines of evidence have revealed a critical role of the hippocampus in the social memory dysfunction associated with ASD [40,41]. In the present study, postnatal Pb exposure in male mice not only led to a notable alteration in the hippocampus, but also significantly induced ASD-like phenotypes such as sociability deficits and repetitive behaviors. These findings further suggest a positive correlation between Pb exposure and the incidence of ASD. In the classic trisynaptic circuit, information proceeds from the entorhinal cortex (EC) to DG, then to CA3, and finally to CA1, which serves as the primary output of the hippocampus. The alterations in the CA2 subregion have received less attention in previous studies. Recent studies have shown the CA2 subregion is crucial for socially cognitive memory [42,43]. Notably, we also observed that, following Pb exposure, the CA2 subregion of the hippocampus exhibited a substantial decrease in neuron numbers and increased morphological abnormalities. These changes may represent potential mechanisms underlying the social deficits by Pb exposure.

ASD is typically diagnosed in early life, mainly before age three—a critical developmental phase of intense synaptogenesis. Disruption of synaptic plasticity is a key mechanism in Pb-induced neuropsychiatric disorders [5,44] and is linked to ASD pathogenesis [45,46]. Our proteomic finding revealed that Pb exposure significantly downregulated the protein expression of DEPs associated with synaptic regulation. This observation further implies a reduction in synaptic signaling, which may be linked to ASD-like behaviors. SYT10 belongs to the SYT family, which mainly participates in the regulation of synaptic vesicle fusion with target plasma membrane. However, the biomolecular functions of SYT10 remain poorly understood. Cao et al. reported that SYT10 functions as the Ca^2+^-sensor, which triggers insulin-like growth factor 1 (IGF-1) exocytosis in olfactory bulb neurons [47]. Deletion of SYT10 impaired activity-dependent IGF-1 secretion, resulting in smaller neurons and an overall decrease in synapse numbers. In a different study, it was demonstrated that the conditional deletion of SYT10 inhibited IGF-1 secretion, thereby preventing socially relevant GABAergic long-term potentiation (LTP) and inducing neuropsychiatric disorders [48,49]. In the current study, employing proteomics and Western blotting analysis, we found that Pb exposure significantly downregulated protein levels of SYT10. Importantly, the protein level of IGF-1 was markedly downregulated in Pb-exposed groups (*p* < 0.05) based on Western blotting analysis. These results suggest that the SYT10/IGF-1 signaling pathway plays important roles in Pb-induced neurodevelopmental toxicity and may also be involved in impairing GABAergic neurotransmission and synaptic plasticity. Nevertheless, further research is needed to elucidate the underlying mechanisms of neurodevelopmental disorders.

In the present study, through STRING analysis and MCL cluster, we identified a cluster closely related to synaptic vesicle function. The cluster pertains to Golgi-associated vesicle biogenesis and the BLOC-1 complex, which consists of biogenesis of lysosome-related organelles complex 1 subunit 4 (BLOC1S4), syntaxin-16 (STX16), and Yip1 domain family member 6 (YIPF6). The BLOC-1 complex, in conjunction with synaptic soluble N-ethylmaleimide-sensitive factor attachment protein receptor (SNARE) proteins, is also proposed to play a role in neurite extension and be involved in intracellular vesicle trafficking [50]. BLOC1S4, a constituent of the biogenesis of lysosome-related organelles complex 1 (BLOC-1), is thought to participate in vesicle-mediated transport and vesicle budding from the trans-Golgi network [51]. STX16, a SNARE protein, was observed to play a key role in regulating polarized neurite growth, particularly dendritic extension [52]. The loss of STX16 function can limit dendritic branching or affect localized transport processes within dendritic trees or spines, thus affecting neural transmission and potentially contributing to detectable behavioral abnormalities [53]. Furthermore, YIPF6 is a five-transmembrane-spanning protein associated with Golgi compartments [54]. It is hypothesized that YIPF proteins play a role in vesicle budding and/or fusion within the Golgi apparatus [55]. These proteins establish a core physical interaction network that is linked to other gene products involved in membrane trafficking, such as SNAREs [55]. The inhibition of the BLOC-1 complex suggested Pb exposure might disrupt the Golgi-associated synaptic vesicle trafficking. The disruption, in turn, could affect neurite extension and ultimately the dysregulation of neural transmission.

In addition to the above-mentioned DEPs, despite the fact that KEGG analysis did not result in its enrichment, we identified a protein, DENN domain-containing protein 2B (DENND2B), which is closely related to synaptic vesicle function. In the Pb exposure groups, DENND2B was significantly downregulated. Notably, Göhring et al. reported that a loss-of-function mutation in DENND2B disrupts vesicle formation and neurotransmitter trafficking [56,57]. This provides further evidence for elucidating that Pb induces developmental disorders by interfering with the function of synaptic vesicles. The dysregulation of synaptic signaling may, in part, account for the molecular pathology of ASD associated with Pb exposure.

Studies have indicated that cell apoptosis may be pivotal in the pathogenesis of ASD [58,59]. In the present study, using KEGG enrichment analysis, the apoptosis pathway was identified, suggesting its key role in ASD-like behaviors by Pb exposure. Pb induced apoptosis not only by reducing the protein level of B-cell lymphoma 2 (Bcl-2), a typical anti-apoptotic marker protein [60], but also by triggering the accumulation of reactive oxygen species (ROS) [61]. Our proteomic analysis demonstrated a critical anti-apoptotic protein, namely Bcl-2-associated athanogene 3 (BAG3), was significantly downregulated by Pb exposure. Its downregulation may imply that neuronal apoptosis was abnormally activated. Moreover, we observed that sodium/myo-inositol cotransporter (SLC5A3) was the only protein that was significantly upregulated in both the 15 mg/kg PbAc and 30 mg/kg PbAc treatment groups. SLC5A3, a member of the solute carrier family 5, is recognized as a sodium-coupled myo-inositol transporter. Functionally, SLC5A3 can positively modulate ROS biosynthesis and oxidative stress responses [62]. Its upregulation suggested that postnatal Pb exposure may also induce ROS accumulation. This, in turn, leads to the abnormal activation of hippocampal neuron apoptosis and triggers the emergence of ASD-like behaviors.

Based on the integrative omics analysis, we have reported that neuroimmune response during development is critically involved in ASD [63]. In the current work, a crucial regulator of immune response, namely Myb-like, SWIRM, and MPN domains containing protein 1 (MYSM1), was identified by proteomic analysis. The deficiency of MYSM1 induced the activation of the NOD2/RIP2 complex [64], and then resulted in excessive inflammation [65]. As for a deubiquitinating enzyme, the deletion of MYSM1 also stimulated PI3K/AKT signaling [66] and exhibited developmental abnormalities [67]. The downregulation of MYSM1 in Pb exposure groups revealed that neuroinflammation may play a key role involved in ASD-like behaviors. Interestingly, through KEGG pathway enrichment, one pathway related with neuroimmune signaling was identified, that is, glycosaminoglycan biosynthesis–chondroitin sulfate/dermatan sulfate. Chondroitin sulfate (CS) serves as a potent inhibitor of the immune response [68,69]. Additionally, it inhibits the activation of human mast cells, and such activation has been associated with ASD [70]. Genetically, reduction in the activity of chondroitin sulfate synthase 1 (CHSS1) leads to neuroinflammation in the mouse hippocampus [71]. Notably, our proteomic analysis found that chondroitin sulfate synthase 2 (CHSS2) was significantly downregulated by Pb exposure, suggesting the possibility of the occurrence of neuroinflammation.

## 5. Conclusions

**Figure 10:**
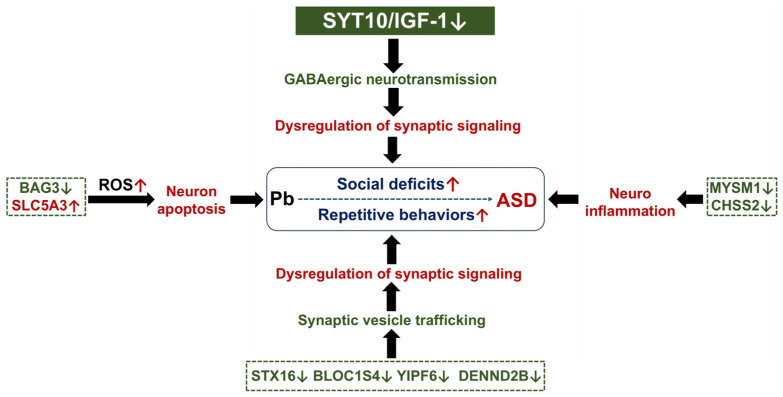
Potential molecular pathology of ASD associated with Pb exposure. White arrow means down-regulation, red arrow means up-regulation, and green arrow means down-regulation.

In summary, we established a mouse model of postnatal Pb exposure, which gave rise to prominent social deficits, repetitive behaviors, and hippocampal neuronal damage. These results clearly demonstrated that postnatal Pb exposure induced autism-like behaviors through alteration of key protein profiling and multi-pathway regulation, including dysregulation of synaptic signaling, abnormal activation of neuron apoptosis, and neuroinflammation (Figure 10). The SYT10/IGF-1 signaling pathway may play a potential key role in Pb-induced neurodevelopmental disorders. However, further research, both in vivo and in vitro, is needed to comprehensively elucidate the mechanism through which Pb disrupts neurodevelopmental integrity. Nevertheless, these findings heighten our understanding of the potential association between environmental heavy metals and neurodevelopmental disorders.

## Data Availability

All relevant data of this study are given in the manuscript. Additional data will be provided upon request.

## Supplementary Materials

The following supporting information can be downloaded at: https://www.mdpi.com/article/10.3390/toxics13060465/s1, Table S1: List of DEPs identified after Pb exposure.

## Abbreviations

ASD	Autism spectrum disorder
SYT10	Synaptotagmin-10
STX16	Syntaxin-16
IGF-1	Insulin-like growth factor 1
