# Supplementary material for: Hippocampal Proteomics Reveals the Novel Molecular Profiling of Postnatal Lead (Pb) Exposure on Autism-like Behaviors
Source: Toxics. 2025 May 31;13(6):465. doi: 10.3390/toxics13060465 (PMC12197489; doi:10.3390/toxics13060465)
Supplement: Supplementary file 1 [file toxics-13-00465-s001.zip › toxics-3640018-supplementary.pdf]

Table S1. List of DEPs identified after Pb exposure

No.	Accessions	Protein name	Gene name	15mg/kg PbAc group *VS* control group		30 mg/kg PbAc group *VS* control group	
				Log2FC	*Q* value	Log2FC	*Q* value
1	Q9JKZ2	Sodium/myo-inositol cotransporter	SLC5A3	0.8741▲	0.0129	0.6866▲	0.0317
2	Q8R4Y8	Rotatin	RTTN	-2.2452▼	0.0019	-2.3363▼	0.0015
3	P51656	17-beta-hydroxysteroid dehydrogenase type 1	HSD17B1	-1.8585▼	0.0034	-2.1258▼	0.0056
4	Q9D979	CHD1 helical C-terminal domain containing protein 1	CHCT1	-1.8327▼	0.0046	-2.0899▼	0.0046
5	Q9R0N4	Synaptotagmin-10	SYT10	-1.8603▼	0.0004	-2.0213▼	0.0001
6	Q924W7	DENN domain-containing protein 2B	DENND2B	-1.9754▼	0.0001	-1.9971▼	0.0000
7	P97313	DNA-dependent protein kinase catalytic subunit	PRKDC	-1.5657▼	0.0003	-1.4836▼	0.0016
8	Q6IEE6	Transmembrane protein 132E	TMEM132E	-1.3076▼	0.0000	-1.3430▼	0.0000
9	Q8CB62	Centrobin	CNTROB	-1.2898▼	0.0061	-1.2643▼	0.0060
10	Q2TA57	Aspartate beta-hydroxylase domain-containing protein 1	ASPHD1	-1.2174▼	0.0001	-1.1286▼	0.0007
11	Q9CYU6	Diphthine methyltransferase	DPH7	-1.0522▼	0.0004	-1.2180▼	0.0035
12	Q8R040	Ribonuclease P protein subunit p21	RPP21	-1.1182▼	0.0133	-1.0834▼	0.0130
13	Q9CZU4	GTPase Era, mitochondrial	ERAL1	-0.9871▼	0.0144	-1.0599▼	0.0051
14	Q8BGT0	Osteopetrosis-associated transmembrane protein 1	OSTM1	-0.9762▼	0.0029	-0.9750▼	0.0095
15	Q6X7S9	EP300-interacting inhibitor of differentiation 2	EID2	-0.8731▼	0.0001	-0.8474▼	0.0002
16	P70662	LIM domain-binding protein 1	LDB1	-0.8132▼	0.0212	-0.8523▼	0.0325
17	Q8VED2	Biogenesis of lysosome-related organelles complex 1 subunit 4	BLOC1S4	-0.8272▼	0.0017	-0.9404▼	0.0009

18	O70472	Transmembrane protein 131	TMEM131	-0.8122▼	0.0002	-0.7732▼	0.0001
19	Q9JLV1	Bcl-2-associated athanogene 3	BAG3	-0.7405▼	0.0010	-0.8209▼	0.0007
20	Q8K2I4	Beta-mannosidase	MANBA	-0.8607▼	0.0000	-0.8865▼	0.0007
21	Q8BVI5	Syntaxin-16	STX16	-0.7198▼	0.0005	-0.7137▼	0.0000
22	Q9Z2B5	Eukaryotic translation initiation factor 2-alpha kinase 3	EIF2AK3	-0.6725▼	0.0036	-0.8529▼	0.0062
23	Q9DBB5	Eukaryotic translation initiation factor 4E type 3	EIF4E3	-0.7080▼	0.0012	-0.7287▼	0.0029
24	Q8C5D8	E3 SUMO-protein ligase PIAS2	PIAS2	-0.7953▼	0.0022	-0.6877▼	0.0004
25	Q9QXK2	E3 ubiquitin-protein ligase RAD18	RAD18	-0.6993▼	0.0111	-0.6047▼	0.0014
26	Q8BWR4	Ubiquitin carboxyl-terminal hydrolase 40	USP40	-0.6572▼	0.0133	-0.7105▼	0.0066
27	Q9CX53	Gem-associated protein 6	GEMIN6	-0.6287▼	0.0003	-0.6927▼	0.0004
28	Q6IQX7	Chondroitin sulfate synthase 2	CHSS2	-0.7005▼	0.0003	-0.8282▼	0.0001
29	Q69Z66	Deubiquitinase MYSM1	MYSM1	-0.8277▼	0.0061	-0.8067▼	0.0028
30	Q5SX40	Myosin-1	MYH1	-0.7630▼	0.0003	-0.6982▼	0.0020
31	Q52KP5	NXPE family member 4	NXPE4	-0.7901▼	0.0017	-0.9128▼	0.0001
32	Q3T9M1	Sphingosine-1-phosphate transporter MFSD2B	MFSD2B	-0.9244▼	0.0000	-0.7632▼	0.0002
33	P13405	Retinoblastoma-associated protein	RB1	-0.9886▼	0.0103	-0.7307▼	0.0352
34	E9PYK3	Protein mono-ADP-ribosyltransferase PARP4	PARP4	-0.7177▼	0.0009	-0.7650▼	0.0057
35	Q9Z127	Large neutral amino acids transporter small subunit 1	LAT1	0.1149●	0.5302	0.7042▲	0.0121
36	Q9DBS1	Transmembrane protein 43	TMM43	0.3029●	0.2022	0.6381▲	0.0415
37	A2AFE9	Proline-rich protein 32	PRR32	0.1399●	0.4950	-2.4433▼	0.0050
38	P02301	Histone H3.3C	H3C	0.0800●	0.6824	-1.3435▼	0.0003

39	P68433	Histone H3.1.	H31	0.0806●	0.6783	-1.3229▼	0.0004
40	Q8BR70	Protein YIPF6	YIPF6	-0.1927●	0.4429	-1.1636▼	0.0485
41	O70433	Four and a half LIM domains protein 2	FHL2	-0.0711●	0.5473	-1.0316▼	0.0107
42	P49138	MAP kinase-activated protein kinase 2	MAPK2	-0.2327●	0.2223	-0.9919▼	0.0244
43	Q9CQS8	Protein transport protein Sec61 subunit beta	SC61B	0.0028●	0.9908	-0.9646▼	0.0273
44	P59280	Kelch-like protein 8	KLHL8	-0.0744●	0.5600	-0.9178▼	0.0106
45	P62274	Small ribosomal subunit protein uS14	RS29	0.1404●	0.5351	-0.8102▼	0.0000
46	Q8VBX0	Ankyrin repeat and SOCS box protein 13	ASB13	-0.2384●	0.2998	-0.8101▼	0.0006
47	Q6A037	NEDD4-binding protein 1	N4BP1	-0.5767●	0.0235	-0.6712▼	0.0193
48	Q91V81	RNA-binding protein 42	RBM42	-0.0087●	0.9436	-0.6705▼	0.0093
49	Q5NBY9	POZ (BTB) and AT hook-containing zinc finger 1	PATZ1	-0.3374●	0.0086	-0.6548▼	0.0009
50	P02798	Metallothionein-2	MT2	-0.0900●	0.4524	-0.6277▼	0.0062
51	Q8C0C4	Serine-rich coiled-coil domain-containing protein 1	CCSER1	-0.4108●	0.0159	-0.6178▼	0.0000
52	Q6ZPY7	Lysine-specific demethylase 3B	KDM3B	-0.4102●	0.0344	-0.6112▼	0.0110
53	Q8BGY4	Kelch-like protein 26	KLH26	-0.0863●	0.7270	-0.5881▼	0.0134
54	A2AJL3	FGGY carbohydrate kinase domain-containing protein	FGGY	-0.3821●	0.0031	-0.5856▼	0.0016
55	Q8BQU6	Gap junction gamma-2 protein	CXG2	0.9122▲	0.0173	0.0933●	0.7117
56	P16015	Carbonic anhydrase 3	CAH3	0.8349▲	0.0030	0.6763●	0.2878
57	P23927	Alpha-crystallin B chain	CRYAB	0.8035▲	0.0400	-0.3685●	0.1391
58	P40240	CD9 antigen	CD9	0.7123▲	0.0243	0.4022●	0.1300
59	F6XLV1	Ciliary rootlet coiled-coil protein 2	CRCC2	0.6981▲	0.0239	-0.0554●	0.7744

60	Q60771	Claudin-11	CLD11	0.6704 ▲	0.0477	-0.2647 ●	0.3493
61	Q91VS7	Microsomal glutathione S-transferase 1	MGST1	0.6101 ▲	0.0103	0.4294 ●	0.0421
62	Q9D154	Leukocyte elastase inhibitor A	ILEUA	0.6078 ▲	0.0355	-0.4041 ●	0.1043
63	Q6JPI3	Mediator of RNA polymerase II transcription subunit 13 like	MD13L	-0.6813 ▼	0.0042	-0.4128 ●	0.0176
64	Q923W1	Trimethylguanosine synthase	TGS1	-0.6253 ▼	0.0000	-0.5620 ●	0.0003
65	P09240	Cholecystokinin	CCKN	-0.6022 ▼	0.0038	0.0303 ●	0.7822
66	Q9ESN4	Complement C1q-like protein 3	C1QL3	-0.5922 ▼	0.0302	-0.1960 ●	0.1964

▲ Up-regulated, ▼ Down-regulated, ● Not-significant
